# Stability, Stimuli‐Responsiveness, and Versatile Sorption Properties of a Dynamic Covalent Acylhydrazone Gel

**DOI:** 10.1002/gch2.201800073

**Published:** 2018-11-06

**Authors:** Haobin Fang, Lingyu Chen, Lihua Zeng, Zujin Yang, Jianyong Zhang

**Affiliations:** ^1^ MOE Laboratory of Polymeric Composite and Functional Materials School of Materials Science and Engineering School of Chemical Engineering and Technology Sun Yat‐Sen University Guangzhou 510275 China

**Keywords:** acylhydrazone, adsorption, dynamic covalent chemistry, gels

## Abstract

Gel adsorbents are promising for pollutant removal from the wastewater. Herein, an acylhydrazone gel is developed from acylhydrazide‐terminated pentaerythritol (PAT) and 2,4,6‐triformylphloroglucinol (TFP) based on dynamic covalent acylhydrazone chemistry. PAT‐TFP gel is stable under various conditions, while it shows reversible Cu^2+^ adsorption and desorption. PAT‐TFP gel is studied as a versatile adsorbent for the capture of a range of (bulky) organic contaminants and heavy metal ions from aqueous solutions. Fast and good adsorption capacities are achieved for various dyes (rhodamine B and methyl orange), amines (aniline, *p*‐chloroaniline, 4‐methylaniline, and *p*‐aminobenzoic acid), phenols (phenol, 1‐naphthol, *p*‐methylphenol, and bisphenol A), and metal ions (Cu^2+^, Cr^3+^, and Hg^2+^). The maximum adsorption capacity is 107.5 mg g^−1^ for Cu^2+^ and the equilibrium adsorption time is 30 min. PAT‐TFP gel can be regenerated efficiently and used repeatedly.

## 1 Introduction

Water contamination caused by heavy metal ions and organic pollutants from industrial effluents has attracted much attention due to its risk for human beings and environment.^1^ Removing the pollutants from water systems is still an important but challenging task. Nowadays, numerous methods have been proposed for heavy metal and organic pollutants removal, such as chemical precipitation,^2^ ion exchange,^3^ adsorption,^4^, ^5^ membrane filtration,^6^ and electrochemical technologies.^7^ Among them, adsorption offers an efficient, cost‐effective, and easy‐handling method to remove and recover these pollutants from aqueous solutions and wastewaters. In comparison, gels assembled from small molecules have high hierarchical porosity, and are tunable and responsive, thus offering significant advantages over other adsorbents, such as activated carbons, zeolites, and polymeric materials.^8^, ^9^, ^10^ However, the reported gel adsorbents usually show some disadvantages including low mechanical strength, poor stability, and lack of regeneration.^11^, ^12^

**Figure 1:**
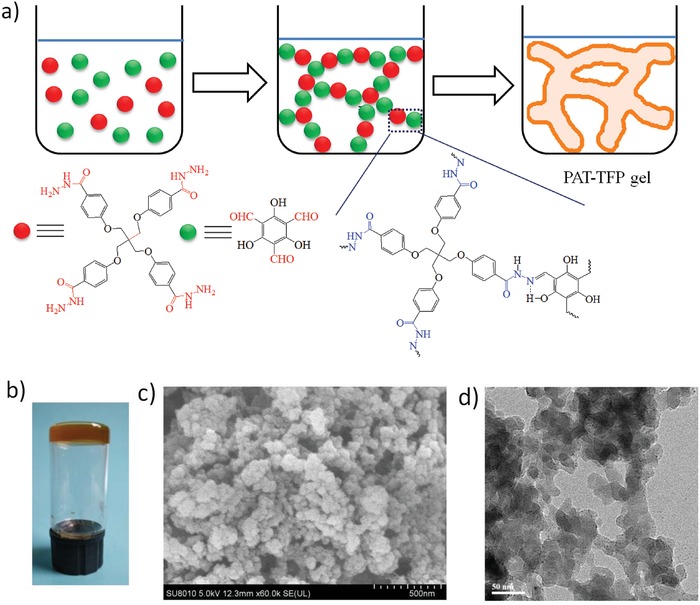
a) Reaction of PAT and TFP to form PAT‐TFP acylhydrazone gel (enol‐imine form), b) photographic image of PAT‐TFP wet gel, c) SEM, and d) TEM images of PAT‐TFP gel (scale bars represent 500 nm for SEM and 50 nm for TEM).

Acylhydrazones provide well‐established ligands for metal ions and organic guests via coordination, H‐bonding, and electrostatic interaction, in view of their chelating capability and structural flexibility. Acylhydrazone formation is a dynamic covalent reaction and has been used for the synthesis of supramolecular physical gels^13^ and polymer gels.^14^ In this work, we develop a novel stable gel based on acylhydrazone chemistry (**Figure** **1**). The acylhydrazone gel is obtained by reacting hydrazide with aldehyde units through dynamic condensation reaction and it shows the following remarkable features. 1) Acylhydrazide‐terminated pentaerythritol (PAT) was chosen because it offers multiple functional ends and is capable of forming hierarchical porous polymeric structures, which are important for mass transfer.^15^ Dynamic covalent polymer gels assembled from small molecules based on acylhydrazone bonding may show high strength.^16^ In addition, its aromatic core may increase the interaction with aromatic organic guests. 2) 2,4,6‐triformylphloroglucinol (TFP) was chosen because its derivatives exhibit rich hydrogen bonding and tautomerism to greatly enhance the materials stability.^17^ 3) Acylhydrazone‐based materials can remove many kinds of metal and organic pollutants.^18^ The resulting PAT‐TFP gel is capable of removal of various organic pollutants, such as dyes, amines and phenols, and metal ions like Cu^2+^, Cr^3+^, Hg^2+^ from aqueous solutions.

## 2 Results and Discussion

Acylhydrazone gels were obtained by reacting PAT with di‐/trialdehydes in DMSO (PAT concentration = 0.03 mol L^−1^, –CONHNH_2_:‐CHO = 1:1) at room temperature (RT) in 1‐2 h. PAT‐TFP gel was prepared from PAT and TFP at RT in ≈2 h to result in an orange translucence gel (Figure 1). For comparison, PAT‐PA acylhydrazone gel was obtained when TFP was replaced with 1,4‐phthalaldehyde (PA) in the presence of HOAc catalyst under similar conditions. SEM and TEM investigation shows that PAT‐TFP has a wormhole‐like porous structure consisting of nanoscale interconnected particles (20–50 nm) with the presence of meso‐ and macropores (Figure 1; Figure S1, Supporting Information). PAT‐TFP is amorphous, as revealed by the broad powder X‐ray diffraction pattern (Figure S2, Supporting Information). FT‐IR spectrum of PAT‐TFP indicates two characteristic bands at 1644 and 1608 cm^−1^ for the ν(C=O) and ν(CH=N) stretching, respectively (Figure S3, Supporting Information).^19^, ^20^, ^21^ The absence of C=C stretching at about 1700–1500 cm^−1^ confirms the existence of enol‐imine form instead of keto‐enamine form.^19^, ^22^ The enol‐imine form of PAT‐TFP was also unambiguously confirmed by ^13^C cross‐polarization magic angle‐spinning (CP‐MAS) solid‐state NMR spectroscopy (Figure S4, Supporting Information). The signal at 146.8 ppm is attributed to the formation of imine C=N bond. The signal is absent at around 180 ppm arising from the carbonyl carbons,^23^, ^24^ which indicates the absence of keto form. The absence of the signal at 192 ppm indicated the total consumption of starting materials. The formation of enol‐imine form is further supported by a control experiment of TFP and benzoylhydrazine PhCONHNH_2_ (Figure S5, Supporting Information). The signal at 8.94 ppm is attributed to the formation of imine bond. ^1^H NMR shows a broad signal at 13.89 ppm corresponding to —OH, indicating the presence of enol‐imine form.^25^

**Figure 2:**
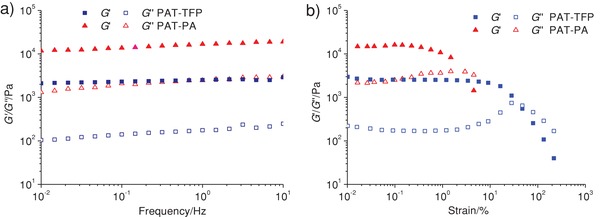
a) Frequency sweep (the strain is 1% for PAT‐TFP and 0.1% for PAT‐PA, and the frequency range is from 0.01 to 10 Hz); b) strain sweep (the frequency is 1 Hz and the strain range is from 0.01% to 100%) for PAT‐TFP and PAT‐PA gels.

Rheological properties were studied for the acylhydrazone gels to assess their behavior under mechanical stress (**Figure** **2**). *G*′ and *G*″ are almost constant with the increase of frequency from 0.01 to 10 Hz. *G*′ is always greater than the corresponding *G*″ within the frequency range 0.01–10 Hz, indicating that the gels are moderately tolerant to external force. The order of *G*′ is PAT‐PA (15000 ± 2000 Pa) > PAT‐TFP (2400 ± 200 Pa), suggesting PAT‐TFP is softer than PAT‐PA^23^ because rigid PA ligand is much easier to form polymeric chains or rigid rods, but flexible TFP ligand tends to be small assemblies.^24^ According to the strain sweep, the order of the upper limit of the linear viscoelastic regime is PAT‐TFP (40%) > PAT‐PA (3%), indicating PAT‐TFP is more stable than PAT‐PA due to the intramolecular hydrogen bond.^26^

PAT‐TFP wet gel is stable at RT in basic or acidic solutions. After PAT‐TFP wet gel was immersed in HCl or NaOH (3 mol L^−1^) for 1 d at RT, FT‐IR spectra did not show significant changes (Figure S3, Supporting Information). In comparison, PAT‐PA gel is also stable at RT in basic solutions, but it collapses in acidic solutions. Thus, the stability of PAT‐TFP is probably attributed to the formation of O—H···N=C intramolecular hydrogen bonding. PAT‐TFP is also stable in the presence of excess salicylic aldehyde or benzaldehyde (40 equiv. based on PAT) below 50 °C for over three weeks. However, the gel collapsed when heated at 100 °C for two weeks, indicating that excess aldehyde can slowly exchange with TFP to break the gel matrix. This confirms that the acylhydrazone bond has reversible formation/hydrolysis and shows capacity to undergo exchange reactions with other aldehydes at higher temperature. But the exchange reaction was hard to go on under mild conditions because of the intramolecular hydrogen bond.^27^

**Figure 3:**
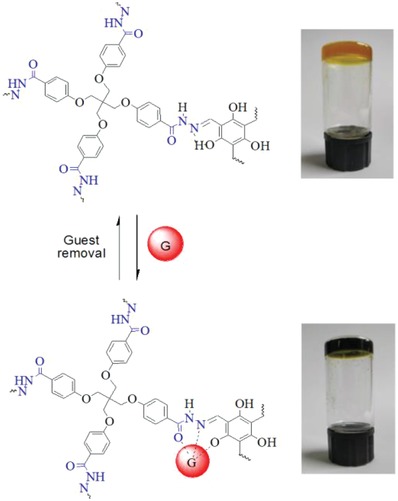
Proposed mechanism of Cu^2+^ adsorption by PAT‐TFP gel and desorption by EDTA. Photographic images of the a) PAT‐TFP gel (top), b) immersed by Cu(NO_3_)_2_ in aqueous solutions (4 equiv. based on PAT) (bottom).

Reversible binding of PAT‐TFP with Cu^2+^ was investigated (**Figure** **3**). As seen, orange PAT‐TFP gel gradually turned to dark green upon the addition of Cu(NO_3_)_2_ aqueous solution (4 equiv.) for 1 d, while the blue Cu^2+^ solution turned colorless due to the strong absorption of Cu^2+^ with acylhydrazone units. Then PAT‐TFP‐Cu gel gradually turned back to orange after addition of disodium EDTA aqueous solution (4 equiv.) due to the stronger binding affinity of Cu^2+^ toward EDTA and the corresponding decomplexation between Cu^2+^ and PAT‐TFP. Rheological measurements of PAT‐TFP and PAT‐TFP‐Cu show that PAT‐TFP gel retains the typical viscoelastic behavior before and after the adsorption of Cu^2+^, evidenced by the higher average storage modulus (*G*′) than the loss modulus (*G*″) (Figure S6, Supporting Information). The critical value of strain for PAT‐TFP‐Cu (26%) is lower than that for PAT‐TFP (33%), indicating that PAT‐TFP‐Cu turns easier to be destroyed probably arising from the weaker intermolecular hydrogen bonding within the acylhydrazone gel matrix after Cu^2+^ adsorption.

The porosity of PAT‐TFP has been characterized by nitrogen physisorption performed at 77 K. Prior to analysis, the wet gels were dried by subcritical CO_2_(l) to get the corresponding aerogels. Thermogravimetric analysis profiles show that PAT‐TFP may remain thermally stable up to ≈270 °C (Figure S7, Supporting Information). The nitrogen isotherms are all closed to type II according to the IUPAC classification (Figures S8 and S9, Supporting Information). They exhibit a rise of uptake within the region *P*/*P*_0_ < 0.01 and a steep rise of uptake within the region *P*/*P*_0_ > 0.9 showing that the micro‐ and macropores are present. The isotherms are accompanied by a hysteresis in the desorption isotherm showing that a range of mesopores are present. The pore size distribution shows a broad size range up to 40 nm estimated by using quenched solid density functional theory (QSDFT). The Brunauer–Emmett–Teller (BET) surface area is 83.4 m^2^ g^−1^ and the pore volume is 0.590 cm^3^ g^−1^ for PAT‐TFP, derived from the adsorption data.

**Figure 4:**
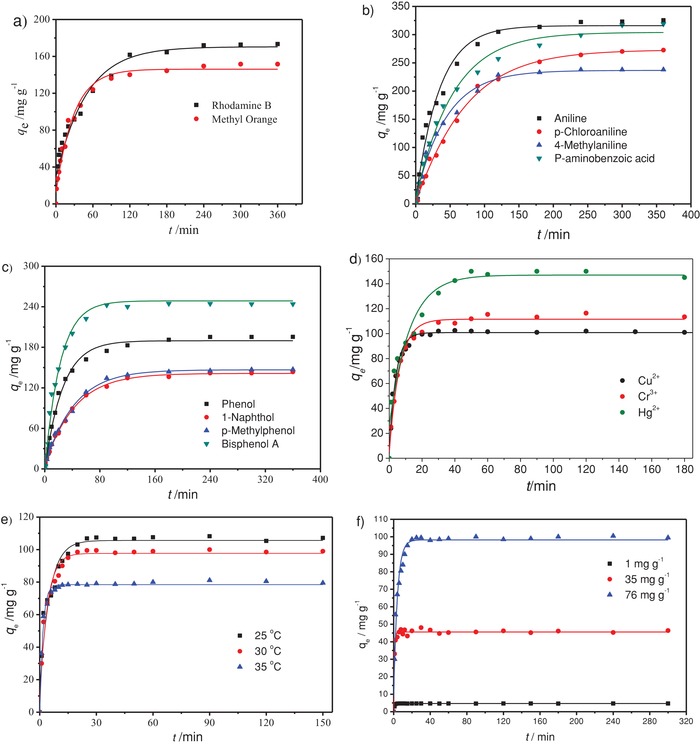
Adsorption kinetic curves of PAT‐TFP aerogel for a) dyes, b) amines, c) phenols, and d) sorption capacity for Cu^2+^, Cr^3+^, and Hg^2+^ at 25 °C (experimental conditions, 100 mL of 100 mg L^−1^ solution at 25 °C and 200 rpm). e) Effect of the contact time and temperature on the adsorption capacity to Cu^2+^ aqueous solution (100 mL, 76 mg L^−1^). f) Sorption capacity for Cu^2+^ with various concentration (1, 35, and 76 mg L^−1^) at 30 °C; 0.020 g of PAT‐TFP aerogel was used for metal ions and 0.010 g for others.

PAT‐TFP aerogel shows removal ability for a wide variety of organic contaminants including dyes, amines, and phenol compounds in aqueous solutions. The adsorption kinetic curves of dyes, amines, and phenol compounds are shown in **Figure** **4**. The adsorption capacities of dyes (cationic rhodamine B and anionic methyl orange) increase rapidly at the beginning, and then gradually attain equilibrium. Adsorption equilibrium is reached in 180 min for rhodamine B and methyl orange. Further increase of contact time has a negligible effect on the equilibrium adsorption capacity. The maximum adsorption capacity for rhodamine B and methyl orange is 173.04 and 151.56 mg g^−1^, respectively. The gel also adsorbs other organic contaminants like amines and phenol compounds. The maximum adsorption capacity for aniline, *p*‐chloroaniline, 4‐methylaniline, and *p*‐aminobenzoic acid is 325.51, 238.02, 272.63, and 319.01 mg g^−1^, respectively, in 300 min. The adsorption equilibrium for phenol, 1‐naphthol, *p*‐methylphenol, and bisphenol A is reached in 320, 340, 100 and 360 min, respectively. The corresponding adsorption capacity was 195.44, 147.53, 143.22 and 244.08 mg g^−1^, respectively. These results suggest that PAT‐TFP has good potentiality for the removal of various organic contaminants from water. The present PAT‐TFP material shows higher adsorption capacity than related Schiff base sorbents like porous organic polymers and gels,^28^ nanohybrid and composite materials,^29^ and covalent organic frameworks^30^ (Table S1, Supporting Information). Moreover, PAT‐TFP absorbs both anionic and cationic dyes^31^ probably due to the electrostatic, hydrogen bonding, and coordinated interaction on the acylhydrazone part.

PAT‐TFP adsorbs various metal ions fast and effectively from aqueous solutions as shown in the adsorption kinetics study (Figure 4). The adsorption capacities of Cu^2+^, Cr^3+^, and Hg^2+^ are 107.5, 115.5, and 150.0 mg g^−1^ after 30, 60, and 50 min, respectively, at 25 °C. The adsorption capacity of PAT‐TFP gel is among the best Schiff base sorbents including inorganic^32^, ^33^ and polymeric materials^34^, ^35^, ^36^ (Table S2, Supporting Information). Furthermore, PAT‐TFP gel needs shorter adsorption time.^33^, ^35^, ^36^ It suggests that PAT‐TFP is a promising sorbent in metal ion removal. To understand the adsorption, adsorption experiments of Cu^2+^ ion were further carried out in detail. The removal efficiency of PAT‐TFP for Cu^2+^ from its 100 mL aqueous solution (76 mg L^−1^) with various temperatures (25, 30, and 35 °C) was investigated (Table S3, Supporting Information). The adsorption capacities increased rapidly at first and then gradually attained equilibrium for 108, 100, and 81 mg g^−1^ in about 30, 25, and 20 min at 25, 30, and 35 °C, respectively. Further increase of contact time had a negligible effect on the equilibrium adsorption capacity. Higher temperature resulted in a quicker equilibrium time due to the faster molecular motion, while resulted in a lower maximal absorption capacity due to the exothermic process during the Cu^2+^ absorption.

The influence of initial metal concentration on the adsorption capacity of PAT‐TFP was studied for concentrations of 1.0, 35, and 76 mg L^−1^ in 100 mL aqueous solution at 30 °C. The maximal absorption capacity was 99.5, 46.9, and 4.65 mg g^−1^ in 25, 8, and 6 min for 1, 35, and 76 mg L^−1^, respectively. When the adsorption was carried out with 1.0 mg L^−1^ solution at 30 °C, the removal rate of Cu^2+^ reached above 93% (4.65 mg g^−1^) in 6 min. It suggests PAT‐TFP may be used in the water sources with very low Cu^2+^ concentration.

The experimental data were evaluated by the pseudo‐first‐order and pseudo‐second‐order kinetic models in order to evaluate the kinetic mechanism (Figure S10, Supporting Information). The correlation coefficient (*R* ^2^) of pseudo‐second‐order model at different temperatures presents higher values (0.999) than those of the pseudo‐first‐order one (<0.96) (Table S4, Supporting Information), which indicates the Cu^2+^ adsorption follows pseudo‐second‐order rate expression and might be controlled by chemical adsorption.^36^

Langmuir, Freundlich, and Dubinin–Radushkevich (D–R) adsorption isotherms are employed to describe the adsorption process of Cu^2+^ onto PAT‐TFP (Figure S11, Supporting Information). The correlation coefficient *R* ^2^ for Langmuir isotherm (0.999) is much higher than that for Freundlich isotherm model (0.956) and is similar with that for D–R adsorption isotherm (0.999) (Table S5, Supporting Information). The maximal absorption capacity is 178.4 mg g^−1^ obtained from Langmuir model. It can be concluded that a monolayer of Cu^2+^ is probably adsorbed onto the surface of PAT‐TFP aerogel.^37^ The apparent adsorption energy is calculated to be 11.33, 11.13, and 11.37 kJ mol^−1^ at 25, 30, and 35 °C, respectively, and it is located between 8 and 16 kJ mol^−1^, meaning that the sorption process occurs chemically. Equilibrium constant for Cu^2+^ adsorption (0.33, 0.34, and 0.40 at 25, 30, and 35 °C, respectively) is in the range of 0 < *R*_L_ < 1, indicating that the uptake of Cu^2+^ onto PAT‐TFP is favorable.^38^ Thermodynamic study reveals the spontaneous nature of Cu^2+^ adsorption indicated by negative *G* ^0^. Increase of *G* ^0^ (−18.52, −18.43, −18.35 kJ mol^−1^) with increasing temperature (25, 30, 35 °C) shows that adsorption process is more favorable at lower temperature (Table S6, Supporting Information). Negative *H* ^0^ (−23.61 kJ mol^−1^) suggests that the adsorption is exothermic in nature. Negative*S* ^0^ (−17.08 J mol^−1^ K^−1^) reveals decreased order at the solid–solution interface during the adsorption on the active sites of the adsorbent.

Regeneration and reusability of adsorbents are important for potential practical applications. To evaluate the reusability of PAT‐TFP aerogel, after sorption, the gel absorbent was regenerated with EDTA (0.24 mol L^−1^) and dried with subcritical CO_2_(l). Atomic absorption spectrometry (AAS) analysis reveals 90% Cu^2+^ was removed from the used gel. The regenerated aerogel was used for Cu^2+^ sorption under the same conditions (concentration 76 mg L^−1^, 25 °C). Repeated sorption–desorption experiments were carried out for consecutive cycles, and the gel could be reused at least three times without significant decrease of the sorption capacity for Cu^2+^.

To probe the sorption mechanism, PAT‐TFP after Cu^2+^ adsorption (PAT‐TFP‐Cu) was investigated. SEM shows that the wormhole‐like porous structure is kept for PAT‐TFP‐Cu (Figure S12, Supporting Information). The presence of Cu atoms is confirmed by energy dispersive X‐ray spectroscopy (EDX), and FT‐IR spectra shows that the ν(C=O) band shifts from 1644 to 1603 cm^−1^ and the ν(—CH=N—) one shifts from 1608 to 1586 cm^−1^, indicating the complexation of Cu^2+^ with C=O and —CH=N— (Figure S13, Supporting Information). X‐ray photoelectron spectroscopy (XPS) measurement of PAT‐TFP and Cu‐PAT‐TFP aerogels demonstrates energy peaks of Cu 2p_3/2_ at 934.3 eV and Cu 2p_1/2_ at 954.5 eV with the shake‐up peaks at 959.1, 944.0, and 940.4 eV (Figure S14, Supporting Information), indicating the presence of Cu^2+^.^39^ Binding energy shifts of N 1s (from 400.6 to 402.3 eV), C 1s (from 286.9 and 288.7 eV to 287.5 and 290.9 eV, respectively), and O 1s (from 533.2 (C=O) and 531.8 eV (OH) to 532.1 and 530.6 eV, respectively) indicate that Cu^2+^ has strong interaction with C=O and C=N as well as OH groups of PAT‐TFP gel.^40^ Therefore, the chemical adsorption of Cu^2+^ onto PAT‐TFP is driven by the complexation between the Cu^2+^ and the N atom in the C=N, O atoms in the C=O and OH.

## 3 Conclusion

In summary, a novel PAT‐TFP gel has been successfully synthesized based on dynamic covalent acylhydrazone chemistry. PAT‐TFP is stable at RT in acidic or basic solutions in contrast to most supramolecular gels due to the formation of O—H···N=C intramolecular hydrogen bonding. PAT‐TFP shows dynamic aldehyde exchange at high temperature, and shows ability to bind reversibly with Cu^2+^. PAT‐TFP gel is effective in the removal of a wide range of (bulky) organic contaminants and harmful metal ions from aqueous solution. PAT‐TFP gel shows fast and good adsorption capacities of various dyes (cationic rhodamine B and anionic methyl orange), amines (aniline, *p*‐chloroaniline, 4‐methylaniline, and *p*‐aminobenzoic acid), phenols (phenol, 1‐naphthol, *p*‐methylphenol, and bisphenol A), and Cu^2+^, Cr^3+^, and Hg^2+^. Detailed investigation of Cu^2+^ adsorption shows that the contact time required to achieve the equilibrium is 30 min, the adsorption process obeys the pseudo‐second‐order kinetics, and the maximal adsorption capacity is 107.5 mg g ^−1^. Furthermore, PAT‐TFP can be regenerated efficiently, and can be used repeatedly for at least three cycles without changing their adsorption capacities for Cu^2+^.

## Conflict of Interest

The authors declare no conflict of interest.

## Supporting information

Supplementary
