# Supplementary material for: Stability, Stimuli‐Responsiveness, and Versatile Sorption Properties of a Dynamic Covalent Acylhydrazone Gel
Source: Glob Chall. 2018 Nov 6;3(2):1800073. doi: 10.1002/gch2.201800073 (PMC6607176; doi:10.1002/gch2.201800073)
Supplement: Supplementary file 1 — Supplementary [file GCH2-3-na-s001.pdf]

Copyright WILEY-VCH Verlag GmbH & Co. KGaA, 69469 Weinheim, Germany, 2018.

Global Challenges
Open Access

Supporting Information

for *Global Challenges,* DOI: 10.1002/gch2.201800073

Stability, Stimuli-Responsiveness, and Versatile Sorption Properties of a Dynamic Covalent Acylhydrazone Gel

Haobin Fang, Lingyu Chen, Lihua Zeng, Zujin Yang, and Jianyong Zhang**

Supporting Information

Stability, stimuli-responsive and versatile sorption properties of a dynamic covalent acylhydrazone gel

Haobin Fang, Lingyu Chen, Lihua Zeng, Zujin Yang, and Jianyong Zhang**

Experimental Section

Infrared spectra were recorded on a Nicolet/Nexus 670 FT-IR spectrometer with KBr pellets in the range 4000-400 cm^{-1}. Scanning electron microscopy (SEM) and energy dispersive X-ray spectroscopy (EDX) were taken using a Quanta 400F scanning electron microscope with an Inca energy dispersive X-ray spectrometer or an Ultra-high Resolution FE-SEM SU8010 scanning electron microscope. Before measurement, the sample was dispersed in ethanol with the aid of sonication, put on aluminum foil, and sputter coated with gold. Transmission electron microscopy (TEM) investigations were carried out on a FEI Tecnai G2 Spirit 120 kV transmission electron microscope. The sample was dispersed in ethanol with the aid of sonication and mounted on a carbon coated copper grid. Powder X-ray diffraction data were collected on a Rigaku SmartLab Powder X-ray diffractometer. Thermo analyses were performed under N_2 atmosphere at a heating rate of 10 K min^{-1} with a NETZSCH TG STA 449 F3 Jupiter system. N_2 adsorption measurements were performed using a Quantachrome Autosorb-iQ2 analyzer. Prior to analysis, the aerogel sample was degassed at 100 ^{o}C for 16 h to remove solvated molecules. NMR spectra were recorded on a Bruker AVANCE 400 Superconducting Fourier Transform Nuclear Magnetic Resonance Spectroscopy instrument. Rheological measurements were carried out on a HAAKE MARS(III) rheometer at 298 K. The diameter of upper plate is 20 mm, and the distance between the lower and upper is 2.6-2.8 mm for tests. X-ray photoelectron spectroscopy (XPS) was tested by X-ray Photoelectron Spectroscopy/ESCA (ESCALAB 250). The contents of dyes, amines and phenols were analyzed by Shimadzu UV-2450 UV-visible spectroscopy. Cu^{2+} and Cr^{3+} contents were analyzed by Hitachi Z-2000 Atomic Absorption Spectrometry (AAS), and Hg^{2+} content was analyzed by Perkin Elmer Optima 8000 Inductively Coupled Plasma- Atomic Emission Spectrometer (ICP-AES).

Preparation of acylhydrazone gels: PAT-TFP gel. Acylhydrazide-terminated pentaerythritol (PAT) (0.0202 g, 0.03 mmol) and triformylphoroglucinol (TFP) (0.0084 g, 0.04 mmol) was each dissolved in DMSO (0.5 mL) respectively. The solutions were mixed at RT and stand for 2 h to obtain an orange translucence gel. The resulting gel was further aged for 24 h.

For subcritical CO_2(l) drying, the gel was subsequently washed with DMSO at RT. DMSO was replaced every day by fresh solvent and solvent exchange was performed for three days. Then the gel was washed with EtOH for three days in similar way. The solvent in the exchanged gel was next extracted with subcritical CO_2(l) (270 g) for 20 h in a 0.75 L high pressure stainless-steel Soxhlet extractor and the extraction temperature was kept at 35 ^{o}C (pressure 5.8 MPa). After the stainless-steel autoclave was depressurized slowly at RT for about 2-3 h to get the PAT-TFP aerogel as orange crushed solid (0.0224 g, 85%).

PAT-PA gel. PAT (0.0202 g, 0.03 mmol) in DMSO (0.5 mL) were mixed with 30 μL acetic acid (3 mol L^{-1} in DMSO) and 1,4-phthalaldehyde (PA) (0.0081 g, 0.06 mmol) in DMSO (0.5 mL) and stand for 1 h at RT to obtain a yellow opaque gel. Using the same extraction process for PAT-TFP, corresponding PAT-PA aerogel was obtained as yellow crushed solid (0.0230 g, 88%).

Adsorption of organic compounds: The adsorption experiments were performed using 100 mg L^{-1} rhodamine B (RB), methyl orange (MO), amine (aniline, *p*-chloroaniline, 4-methylaniline and *p*-aminobenzoic acid), or phenol (phenol, 1-naphthol, p-methylphenol and Bisphenol A) in a temperature-controlled shaking incubator. 0.010 g of PAT-TFP aerogel was added to 100 mL of each solution and the mixture were shaken at 25 ^{o}C and 200 rpm. 1 mL supernatant was taken at intervals, transferred to a 10 mL volumetric flask and diluted with water to the volume. After that, the samples were determined by UV–visible spectroscopy with a definite time interval.

Metal ion sorption and reusability: Adsorption experiments of metal ions were carried out in a 100 mL Erlenmeyer flasks containing 100 mL Cu^{2+}, Cr^{3+}, or Hg^{2+} aqueous solution (concentration 100 mg L^{-1}) in a temperature controlled shaking incubator at different contact times (0-360 min) with 0.020 g aerogel. 100, 76, 35 and 1.0 mg L^{-1} Cu^{2+} aqueous solution was used for the detail study. The flasks were then shaken at 25, 30, or 35 ^{o}C and 200 rpm to reach equilibrium. 1 mL of supernatant was

taken at intervals. The supernatant samples of Cu^{2+} and Cr^{3+} was diluted for 10 times and analyzed by atomic absorption spectrometry (AAS), and the supernatant samples of Hg^{2+} were diluted for 50 times, and analyzed by ICP-AES.

For evaluating reusability, after the sorption of 76 mg L^{-1} Cu^{2+} aqueous solution, the gel was washed by water (100 mL×3) and further desorption was carried out in 0.24 mmol EDTA aqueous solution (100 mL). The mixture was shaken for 10 h at 30 °C and 200 rpm. After desorption, the gel was washed by water (100 mL×3) and EtOH (100 mL×3) in turn. The solvent in the exchanged gel was next extracted with subcritical CO_2(l) (270 g) for 20 h in a 0.75 L high pressure stainless-steel Soxhlet extractor and the extraction temperature was kept at 35 °C (pressure 5.8 MPa). After the stainless-steel autoclave was depressurized slowly at RT for about 2-3 h to get the regenerated aerogel.

The amount adsorbed per unit mass of adsorbent at equilibrium was obtained by using the following equation,

$$q_e = \frac{(C_0 - C_e)V_0}{M}$$

Where q_e (mg g^{-1}) was the adsorption capacity at equilibrium, C_0 and C_e are the initial and equilibrium concentration (mg L^{-1}) of adsorbate respectively, M is the adsorbent dosage (g), and V_0 is the volume of the solution (L).

Adsorption kinetics: The pseudo-first-order model described by the Lagergren equation is presented in the linear form as follow,

$$\ln(q_e - q_t) = \ln q_e - k_1 t$$

where q_e (mg g^{-1}) and q_t (mg g^{-1}) are the amounts of Cu^{2+} sorbed atequilibrium and at time t. k_1 (min^{-1}) is the rate constant of firstorder sorption process. The pseudo-first order kinetic constants were determined from slope of the plot of $\ln(q_e - q_t)$ versus t.

The kinetic data were further analyzed using Ho’s pseudo-second-order kinetic model. The linearized form of the equation is expressed as

$$\frac{t}{q_e} = \frac{1}{k_2 q_e^2} + \frac{t}{q_e}$$

Where q_e and q_t are the amount of the Cu^{2+} removal per unit massof sorbent (mg g^{-1}) at equilibriumand at time *t* (min), and k_2 (g $mg^{-1}min^{-1}$) is the pseudo-second order rate constant. The sorption rate constant (k_2) is obtained from linear plot of t/q_t versus *t*.

Adsorption isotherm: The Langmuir isotherm assumes that the mechanism of the adsorption process is a monolayer on the surface of the adsorbent by the adsorbate. The Langmuir isotherm is based on theassumption that maximum adsorption corresponds to a saturatedmonolayer of solute molecules on the adsorbent surface, that the energy of adsorption is constant

$$\frac{C_e}{q_e} = \frac{C_e}{q_m} + \frac{1}{K_L q_m}$$

where C_e (mg L^{-1}) is the equilibrium solute concentration, q_m (mg g^{-1}) and q_e (mg g^{-1}) is the maximum and equilibriumamount of adsorbate adsorbed per unit mass of adsorbent, K_L is the Langmuir constant (L mg^{-1}) relating the freeenergy of sorption.

The Freundlich isotherm describes the heterogeneous surfaceenergies by multilayer adsorption. The linearized form of theFreundlich equation is given as

$$\ln q_e = \ln K_f + \frac{1}{n} \ln C_e$$

Where K_f (mg g^{-1}) and $1/n$ are the Freundlich constants related to thesorption capacity of the adsorbent and empirical parameter relatingthe adsorption intensity, respectively

The D-R model has been used to describe the sorption of Cu^{2+} and is represented as follow,

$$\ln q_e = \ln q_m - K\varepsilon^2$$

$$\varepsilon = RT\ln\left(1 + \frac{1}{C_e}\right)$$

$$E = \frac{1}{\sqrt{2k}}$$

where ε is the Polanyi potential, where R and T are the universal gas constant (kJ mol^{-1} K^{-1}) and temperature (^{o}C), respectively.

The degree of suitability of adsorbent toward heavy metal ions can be described by equilibrium constant R_L, which can be calculated from the following equation,

$$R_L = \frac{1}{1 + K_L C_0}$$

where C_0 is the initial concentration of metal ions (mg L^{-1}) and K_L is the Langmuir constant.

The Gibbs free energy change, G^0, is the fundamental criterion of spontaneity. The standard Gibbs free energy was computed atdifferent temperatures according to the following equation,

$$\Delta G^0 = \Delta H^0 - T\Delta S^0$$

The enthalpy (H^0) and entropy (S^0) change values were estimated from the following equation,

$$\ln K_L = -\frac{\Delta H^0}{RT} + \frac{\Delta S^0}{R}$$

where, R is the universal gas constant (kJ mol^{-1} K^{-1}), the temperature (T, ^{o}C) and K_L is obtained by multiplying Langmuir constant. The changes in enthalpy (H^0) and entropy (S^0) were estimated from the slope and intercept of the plot of ln K_L versus -1/T.

a) 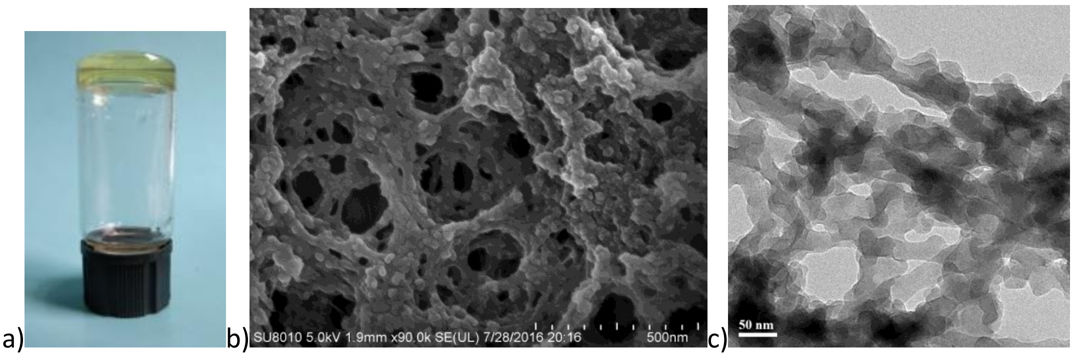

Figure S1. a) Photographic image of PAT-PA wet gel, b) SEM and c) TEM images of PAT-PA gel (scale bar represents 500 nm for SEM and 50 nm for TEM).

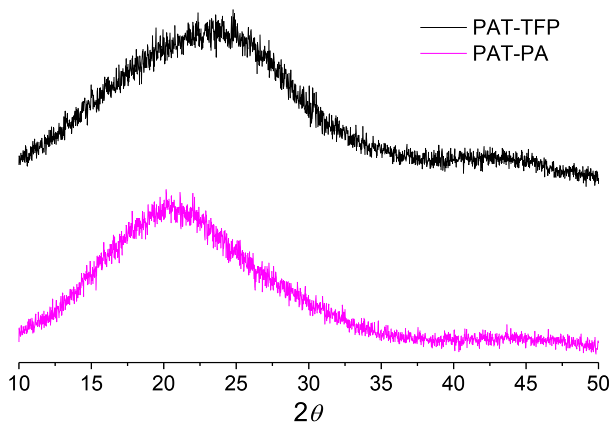

Figure S2. Powder X-ray diffraction patterns of PAT-TFP and PAT-PA aerogels.

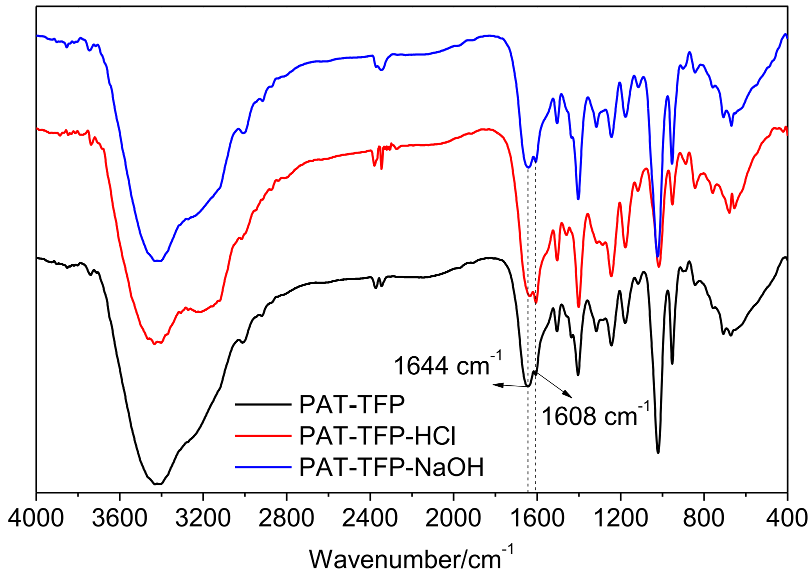

Figure S3. FT-IR spectra of a) PAT-TFP gel (solvent was exchanged into water), and the gel after immersing in b) HCl or c) NaOH aqueous solution (3 mol L^{-1}) at RT for 1 d.

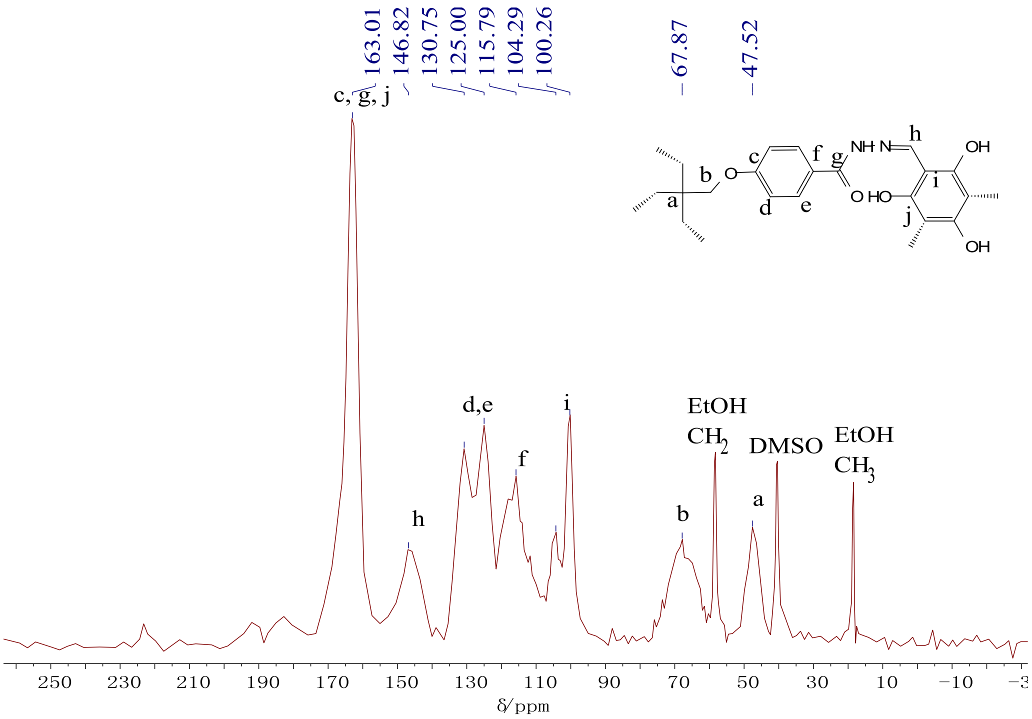

Figure S4. Solid-state ^{13}C CP/MAS NMR spectrum of PAT-TFP aerogel (400 MHz).

^{13}C solid-state NMR (400 MHz): 163.0 (c, g, j), 146.8 (h), 130.8, 125.0 (d, e), 115.8 (f), 104.3, 100.3 (i), 67.9 (b), 47.5 (a). The signals at 58.3 and 18.5 ppm are assigned to residual EtOH. The signal at 40.0 ppm is assigned to residual DMSO.

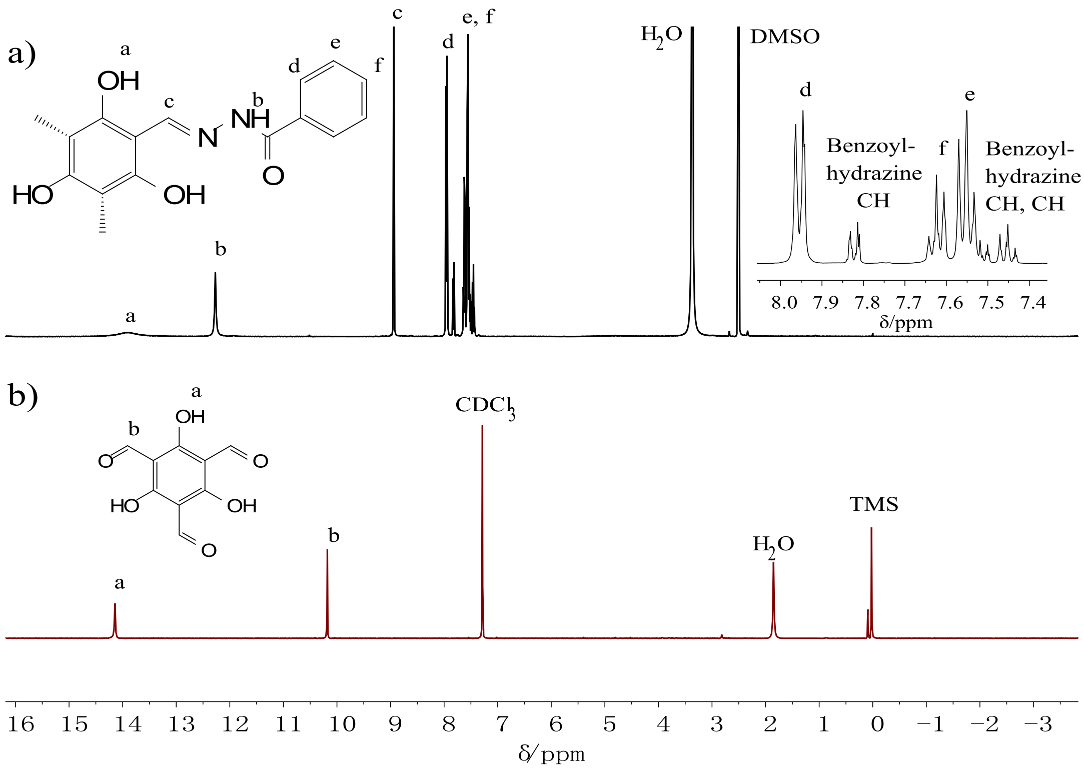

Figure S5. ^{1}H NMR spectra (400 MHz, 298 K) of a) reaction mixture of TFP and benzoylhydrazine in DMSO-d_6 (1:4, c_{TFP} = 0.04 mol L^{-1}) at RT for 1 d, and b) TFP in DMSO-d_6.

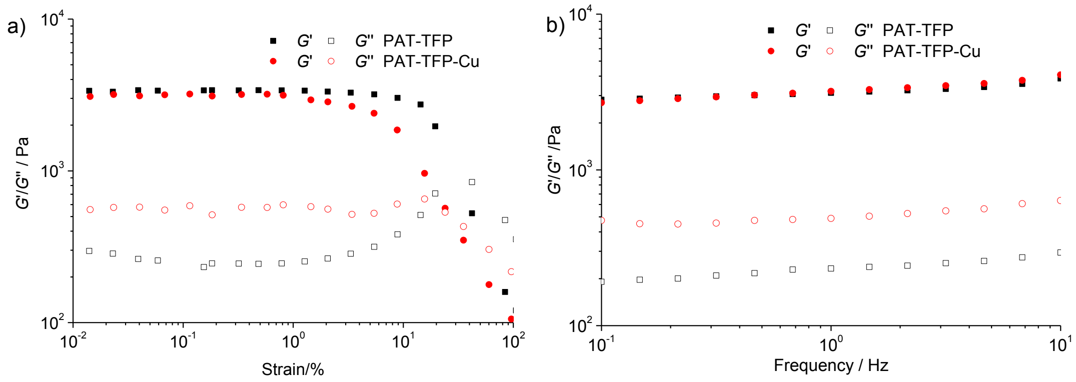

Figure S6. Rheological tests of PAT-TFP and PAT-TFP-Cu gels, a) strain sweep at constant frequency of 1 Hz and b) frequency sweep at constant strain of 1%.

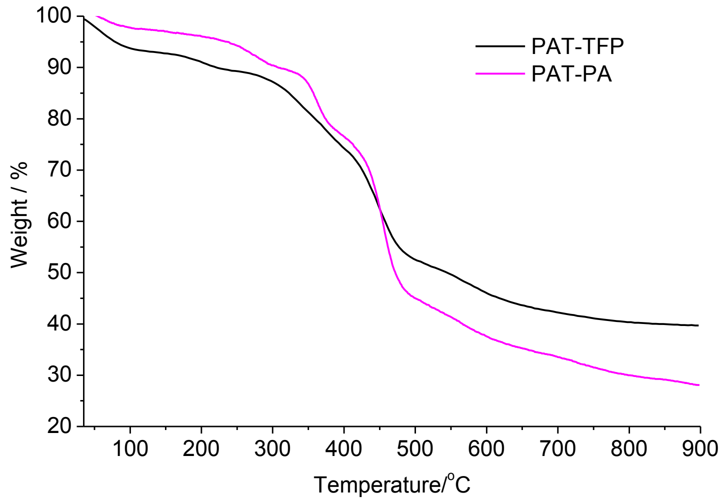

Figure S7. Thermogravimetric analysis profiles of PAT-TFP and PAT-PA aerogels under N_2 atmosphere.

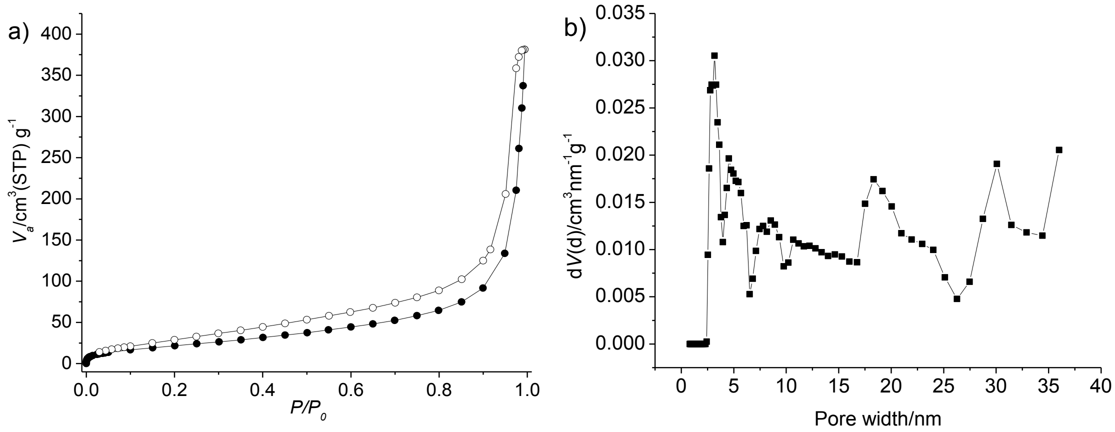

Figure S8. N_2 adsorption (closed symbols) /desorption (open symbols) isotherms for PAT-TFP aerogel at 77 K; b) quenched solid DFT pore size distribution (model: N_2 at 77 K on carbon, cylindr./sphere pores, QSDFT adsorption branch).

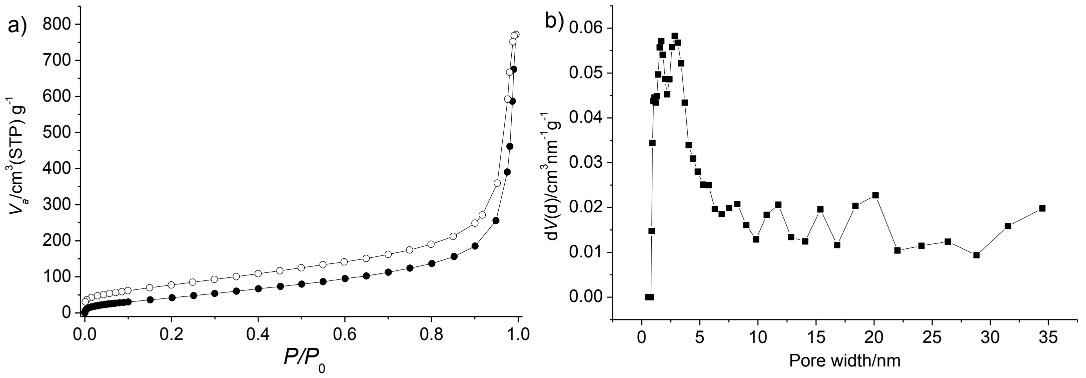

Figure S9. N_2 adsorption (closed symbols) /desorption (open symbols) isotherms for PAT-PA aerogel at 77 K; b) quenched solid DFT pore size distribution (Model: N_2 at 77 K on carbon, cylindr./sphere pores, QSDFT adsorption branch).

Table S1. Comparison of the adsorption capacity of PAT-TFP aerogel with those of related Schiff base sorbents for dyes, amine and phenol compounds.

Adsorbent	Absorbate[a]	q_{max}/mg g^{-1}	t_e /min	Ref.
Schiff base porous organic polymer from melamine and terephthalaldehyde	MO	140.2 (pH 7)	240	1
chitosan/polyacrylonitrile Semi-IPN hydrogel	RB	17.5	240	2
polymers of intrinsic microporosity (PIM-1) electrospun ultrafine fibers	aniline	161.2	300	3
Chitosan modified by a cross-linked β-cyclodextrin polymer (EPI-CD)	phenol	131.5	180	4
PP-g-DMAEMA/PM (polymer of dimethylaminoethyl methacrylate with modification of porous microspheres on the surface of polypropylene fiber	Bisphenol A	44.43	40	5
β-cyclodextrin-carboxymethylcellulose-based hydrogels	Bisphenol A	38.1	300	6
nanohybrid material containing a Cd(II) semicarbazone Schiff base complex and phosphomolybdic acid	RB<br>MO	35.8<br>~0	6	7
Mn-nanoparticles (MnNPs) supported on the Schiff base modified nano-sized SiO_2-Al_2O_3 mixed-oxides	MO	145.7	45	8
Montmorillonites modified by bispyridinium dibromides	phenol<br>p-methylphenol	42.8<br>＜42.8	120<br>120	9

Azine-linked covalent organic framework	Bisphenol A	61.3	5	10
CuP-DMNDA-COF/Fe (imine-linked porphyrin covalent organic framework)	RB	378.00	≈ 400	11

[a] RB, rhodamine B; MO, methyl orange

[1] H. Ou, Q. You, J. Li, G. Liao, H. Xia, D. Wang, *RSC Adv.* **2016**, *6*, 98487-98497.

[2] F. S. Al-Mubaddel, S. Haider, M. O. Aijaz, A. Haider, T. Kamal, W. A. Almasry, M. Javid, S. U. Khan, *Polym. Bull.* **2017**, *74*, 1535-1551.

[3] B. Satilmis, T. Uyar, *J. Colloid Interface Sci.* **2018**, *516*, 317-324.

[4] J. -M. Li, X. -G. Meng, C. -W. Hu, J. Du, *Bioresour. Technol.* 2**009**, *100*, 1168-1173.

[5] L. Cui, J. Wei, X. Du, X. Zhou, *Ind. Eng. Chem. Res.* **2016**, *55*, 1566-1574.

[6] H. Kono, K. Onishi, T. Nakamura, *Carbohydr. Polym.* **2013**, *98*, 784-792.

[7] S. Farhadi, M. M. Amini, M. Dusek, M. Kucerakova, F. Mahmoudi, *J. Mol. Struct.* **2017**, *1130*, 592-602

[8] M. Arshadi, F. Mousavinia, M. J. Amiri, A. R. Faraji, *J. Colloid Interface Sci.* **2016**, *483*, 118-131

[9] Z. Luo, M. Gao, S. Yang, Q. Yang, *Colloids Surf. A: Physicochem. Eng. Aspects* **2015**, *482*, 222-230.

[10] Z. Liu, H. Wang, J. Ou, L. Chen, M. Ye, *J. Hazard. Mater.* **2018**, *355*, 145-153.

[11] Y. Hou, X. Zhang, C. Wang, D. Qi, Y. Gu, Z. Wang, J. Jiang, *New J. Chem.* **2017**, *41*, 6145-6151.

Table S2. Comparison of the adsorption capacity of PAT-TFP gel with those of related Schiff base sorbents for metal ions.

Adsorbent	Metal ion	q_{max}/mg g^{-1}	t_e/min	Ref.
Silica gel functionalized with ditopic zwitterionic Schiff base ligand	Cu^{2+}	41.31	60	12
Silica gel modified with ditopic zwitterionic Schiff base	Cu^{2+}	26.88	90	13
Silica particles with Schiff base receptor	Hg^{2+}	99.0	30	14
SB-SiO_2@MNPs (silica based magnetic nanoparticles with Schiff base moiety)	Cu^{2+}	13.25	25	15
MNS2/MN3 (Schiff base-modified nanoparticles)	Cu^{2+}	4.12/5.92	20/50	16
SBA/En & SBA/EnSA (SBA-15 particles bearing ethylenediaminopropyl and ethylenediaminepropylesalicylaldimine ligands	Hg^{2+}	6.3/8.3	30/30	17
MCM-41-NH_2/MCM-41-N-Hdhba (MCM-41 modified with Schiff base)	Cu^{2+}	138.80/222.20	40/60	18
poly(MVE-alt-MA-1) resin.	Cu^{2+}	81.72	≈ 240	19
	Cr^{3+}	29.70	≈ 240	
Gu-MC (guanyl-modified cellulose)	Cu^{2+}	83	100	20
	Hg^{2+}	48	100	
m-ECCSB (magnetic-epichlorohydrin crosslinked chitosan Schiff's base)	Cu^{2+}	123.10	60	21
Hydroxyl amine modified polyacrylonitrile	Cu^{2+}	105	240	22

micro/nanofibers	Cr^{3+}	204	240

[12] Q. Wang, W. Gao, Y. Liu, J. Yuan, Z. Xu, Q. Zeng, Y. Li, M. Schröder, *Chem. Eng. J.* **2014**, *250*, 55-65.

[13] Z. Xua, K. Wang, Q. Liu, F. Guo, Z. Xiong, Y. Li, Q. Wang, *Sep. Purif. Technol.* **2018**, *191*, 61-74.

[14] S. Radi, Y. Toubi, M. Bacquet, S. Degoutin, Y. N. Mabkhot, Y. Garcia, *RSC Adv.* **2016**, *6*, 34212-34218.

[15] R. Mutneja, R. Singh, V. Kaur, J. Wagler, S. Fels, E. Kroke, *New J. Chem.* **2016**, *40*, 1640-1648.

[16] M. K. Moftakhar, M. R. Yaftian, M. Ghorbanloo, *Int. J. Environ. Sci. Technol.* **2016**, *13*, 1707-1722.

[17] M. H. Dindar, M. R. Yaftian, M. Hajihasani, S. Rostamnia, *J. Taiwan Inst. Chem. Eng.* **2016**, *67*, 325-337.

[18] E. M. Saad, H. M. A. Hassan, M. S. Soltan, I. S. Butler, S. I. Mostafa, *Environ. Prog. Sustain. Energy* **2017**, *37*, 746-760.

[19] M. Cegłowski, G. Schroeder, *Chem. Eng, J.* **2015**, *263*, 402-411.

[20] I. M. Kenawy, M. A. H. Hafez, M. A. Ismail, M. A. Hashem, *Int. J. Biol. Macromol.* **2018**, *107*, 1538-1549.

[21] Y. Gutha, Y. Zhang, W. Zhang, X. Jiao, *Int. J. Biol. Macromol.* **2017**, *97*, 85-98.

[22] H. Lou, X. Cao, X. Yan, L. Wang, Z. Chen, *Water Sci. Technol.* **2017**, *2*, 378-389.

Table S3. Experimental data for the contact time and temperature on maximal adsorption capacity of PAT-TFP aerogel to Cu^{2+} aqueous solution (100 mL, 76 mg L^{-1}) (repeated for 3 times).

T/°C	1		2		3	
	q_e/ mg g^{-1}	t_e /min	q_e/ mg g^{-1}	t_e /min	q_e/ mg g^{-1}	t_e /min
25	102.0	30	106.1	25	107.5	30
30	96.8	20	99.5	25	94.3	20
35	75.8	15	78.8	20	79.1	20
40	56.4	10	61.4	12	60.5	12

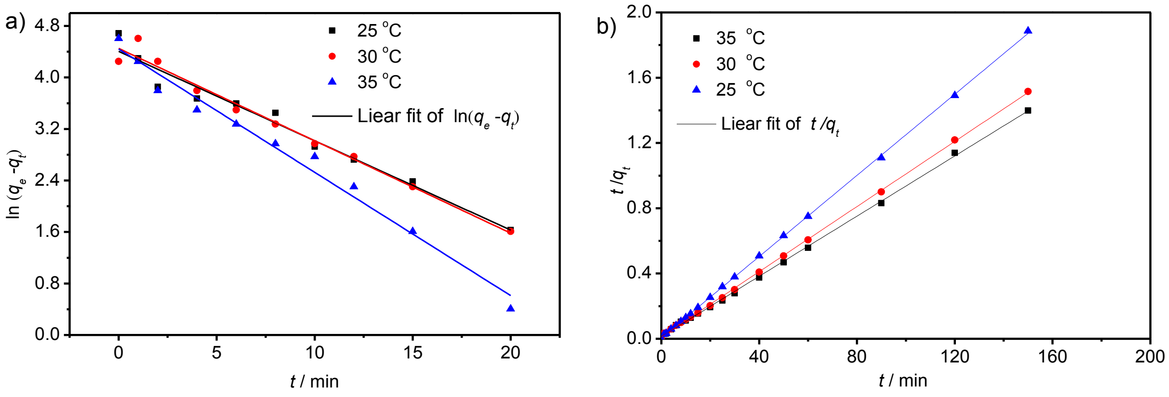

Figure S10. Linear kinetic plots for the Cu^{2+} adsorption on PAT-TFP aerogel, a) pseudo-first-order and b) pseudo-second-order model.

Table S4. Kinetic parameters for the Cu^{2+} adsorption on PAT-TFP aerogel at vaious temperature.

model	parameter	*T*/°C		
		25	30	35
pseudo-first-order	C_0/mg L^{-1}	76	76	76
	$q_{e,exp}$/mg g^{-1}	108.25	100	81.15
	k_1/min^{-1}	0.138	0.143	0.191
	$q_{e,cal}$/mg g^{-1}	81.84	85.78	85.00
	R^2	0.935	0.956	0.959
pseudo-second-order	k_2/g mg^{-1} min^{-1}	0.006045	0.008305	0.02365
	$q_{e,cal}$/mg g^{-1}	108.69	101.01	80.64
	R^2	0.999	0.999	0.999

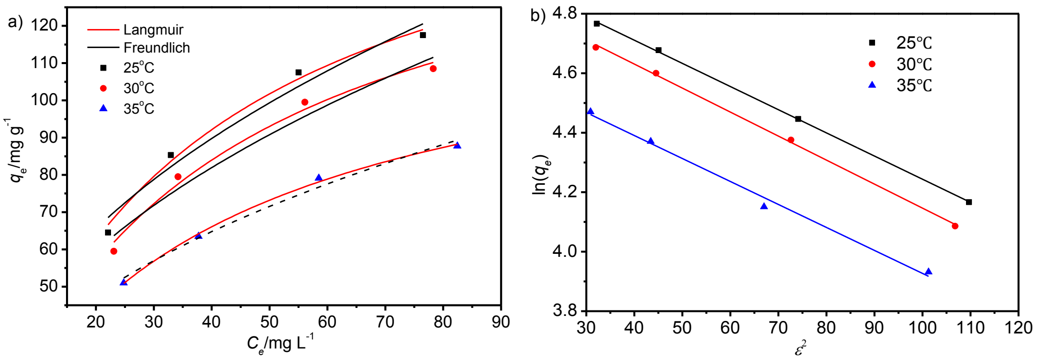

Figure S12. a) Isotherm plots of Langmuir isotherm and Freundlich isotherm, b) isotherm plots of Dubinin-Radushkevich (D-R) isotherm for the Cu^{2+} adsorption on PAT-TFP aerogel.

Table S5. Correlated parameters for the Cu^{2+} adsorption on PAT-TFP aerogel from aqueous solution according to Langmuir, Freundlich, and D-R models.

parameter	*T*/°C		
	25	30	35
	Langmuir model		
q_m/ mg g^{-1}	178.4	166.66	153.59
K_L/ L mg^{-1}	0.0266	0.0252	0.0195
R^2	0.999	0.999	0.989
R_L	0.33	0.34	0.40
	Freundlich model		
K_f/ L mg^{-1}	15.13	13.38	10.14
1/n	0.48	0.49	0.52
R^2	0.956	0.946	0.967
	D-R model		
q_m/ mg g^{-1}	151.41	141.17	109.95
K	0.00779	0.00807	0.00773
E/ kJ mol^{-1}	11.33	11.13	11.37
R^2	0.999	0.999	0.989

Table S6. Thermodynamic parameters for the Cu^{2+} adsorption on PAT-TFP aerogel.

T/°C	ln K_L	Δ*G*° /kJ mol^{-1}	Δ*H*° /kJ mol^{-1}	Δ*S*° /J mol^{-1} K^{-1}
25	7.44	-18.52		
30	7.39	-18.43	-23.61	-17.08
35	7.13	-18.35		

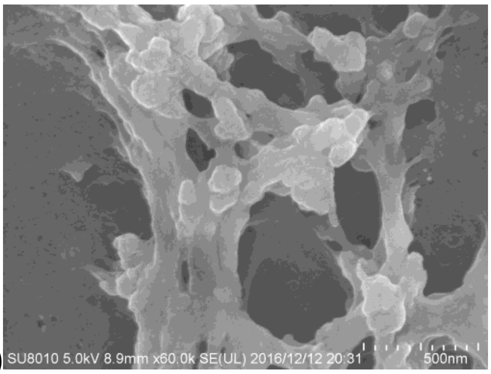

a)

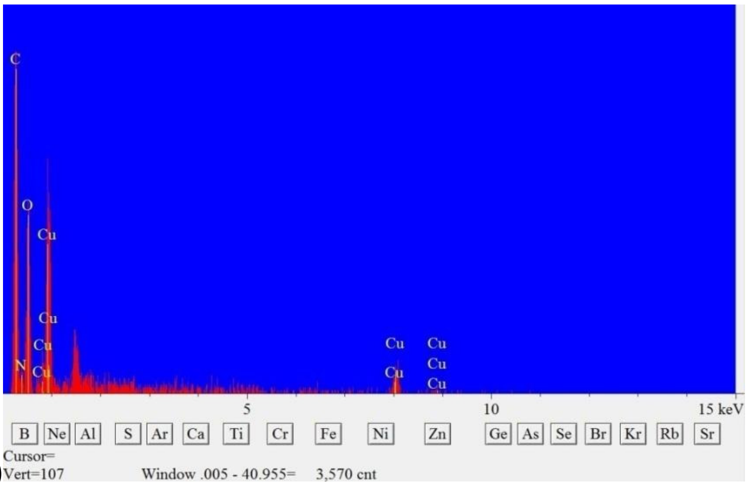

b)

Figure S12. a) SEM image (scale bar represents 500 nm), and b) EDX spectrum (quantitative value/atom%, Cu 3.328) of PAT-TFP-Cu.

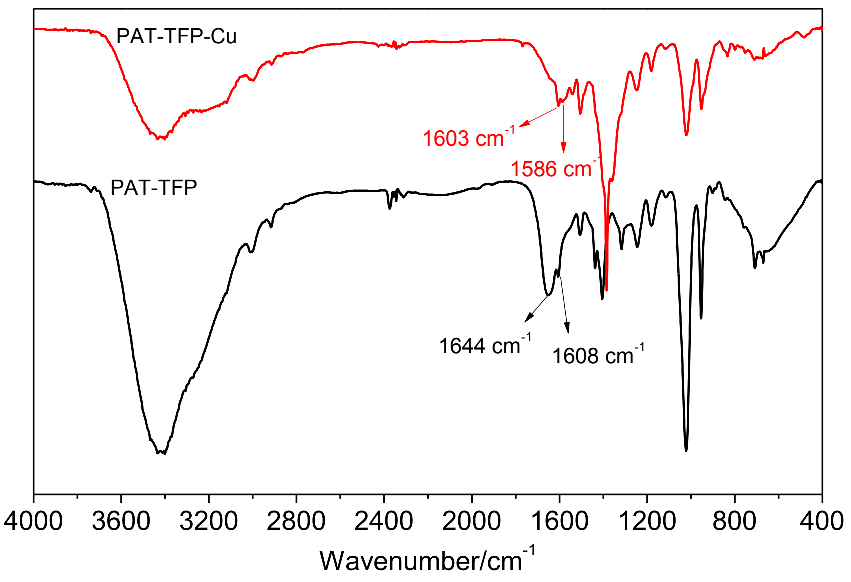

Figure S13. FT-IR spectra of PAT-TFP and PAT-TFP-Cu wet gels (solvent was exchanged into water) before and after Cu^{2+} adsorption, respectively.

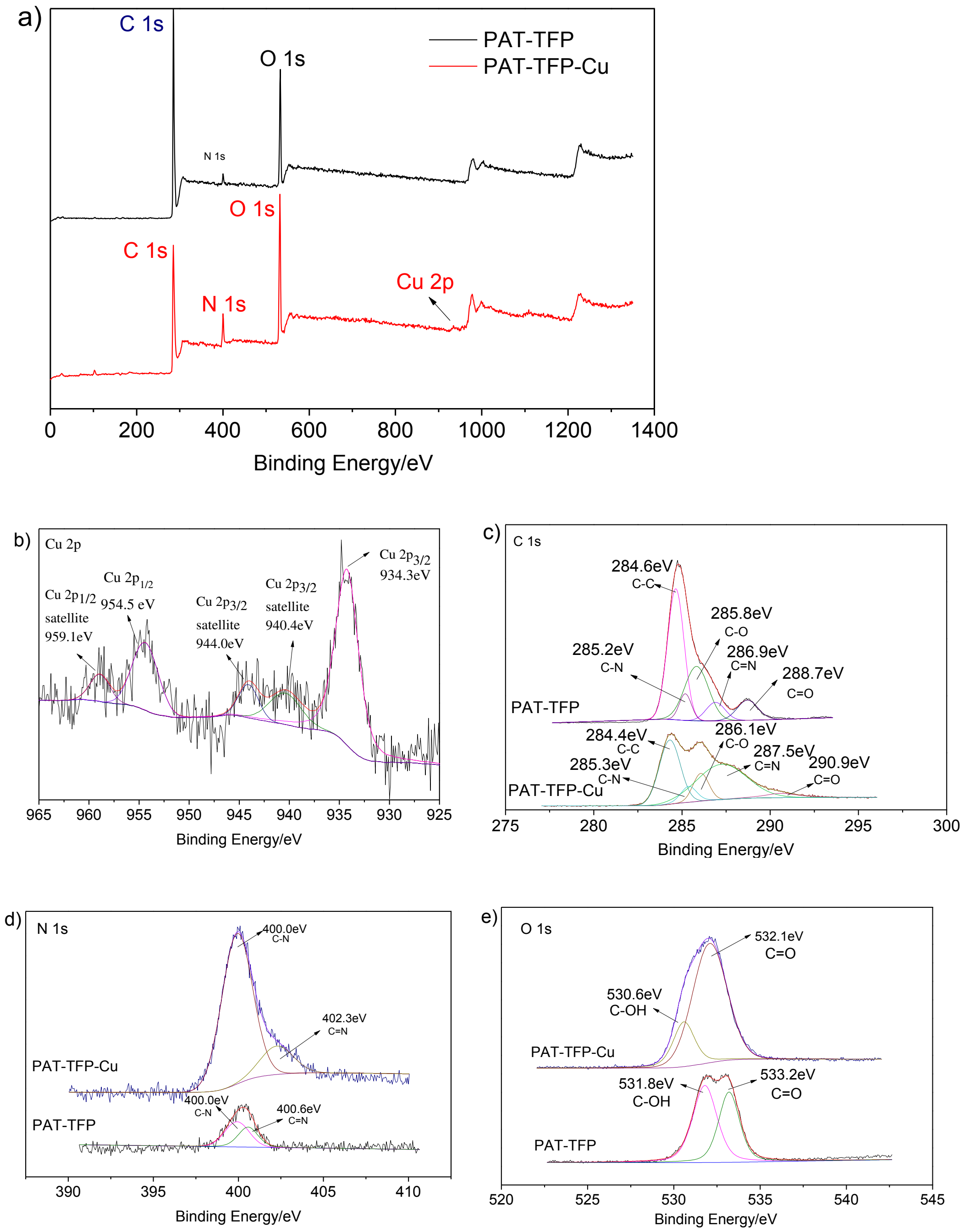

Figure S14. X-ray photoelectron spectroscopy, a) full scan, b) Cu 2p, c) C 1s, d) N 1s and e) O 1s spectra of PAT-TFP and PAT-TFP-Cu before and after Cu^{2+} adsorption, respectively.
